# Hybrid Twins Modeling of a High-Level Radioactive Waste Cell Demonstrator for Long-Term Temperature Monitoring and Forecasting

**DOI:** 10.3390/s24154931

**Published:** 2024-07-30

**Authors:** David Muñoz, Anoop Ebey Thomas, Julien Cotton, Johan Bertrand, Francisco Chinesta

**Affiliations:** 1 PIMM Laboratory, Arts et Métiers Institute of Technology, Centre National de la Recherche Scientifique (CNRS), 151 Boulevard de l’Hôpital, 75013 Paris, France; francisco.chinesta@ensam.eu; 2 ESI Group, Symbiose 2, 10 Avenue Aristide Briand, 92220 Bagneux, France; anoopebey.thomas@esi-group.com; 3 Andra, French National Radioactive Waste Management Agency, 92298 Châtenay-Malabry, France; julien.cotton@andra.fr (J.C.); johan.bertrand@andra.fr (J.B.)

**Keywords:** temperature forecast, high-level radioactive waste management, hybrid twins, parametric modeling, LSTM cells

## Abstract

Monitoring a deep geological repository for radioactive waste during the operational phases relies on a combination of fit-for-purpose numerical simulations and online sensor measurements, both producing complementary massive data, which can then be compared to predict reliable and integrated information (e.g., in a digital twin) reflecting the actual physical evolution of the installation over the long term (i.e., a century), the ultimate objective being to assess that the repository components/processes are effectively following the expected trajectory towards the closure phase. Data prediction involves using historical data and statistical methods to forecast future outcomes, but it faces challenges such as data quality issues, the complexity of real-world data, and the difficulty in balancing model complexity. Feature selection, overfitting, and the interpretability of complex models further contribute to the complexity. Data reconciliation involves aligning model with in situ data, but a major challenge is to create models capturing all the complexity of the real world, encompassing dynamic variables, as well as the residual and complex near-field effects on measurements (e.g., sensors coupling). This difficulty can result in residual discrepancies between simulated and real data, highlighting the challenge of accurately estimating real-world intricacies within predictive models during the reconciliation process. The paper delves into these challenges for complex and instrumented systems (multi-scale, multi-physics, and multi-media), discussing practical applications of machine and deep learning methods in the case study of thermal loading monitoring of a high-level waste (HLW) cell demonstrator (called ALC1605) implemented at Andra’s underground research laboratory.

## 1. Introduction

Measuring devices, data processing, and numerical simulation are three fundamental components for monitoring complex installations or systems such as geological radioactive waste repositories. They facilitate the quantification of diverse physical parameters (including THMC for Thermal, Hydro, Mechanical, and Chemical parameters) and their evolution over extended periods. Successive European projects like MODERN2020 and MODATS have pooled international expertise in this field for almost a decade, concluding that measurement technologies exist for all monitoring parameters despite varying levels of technological readiness. Data processing is essential for handling, analyzing, and interpreting collected information, supporting decision-making, troubleshooting, and understanding of studied phenomena. Technological advancements have improved the precision, compactness, and accessibility of measuring devices, while data processing methods have evolved to meet increasing demands for data analysis and interpretation. Integrating digital simulations with online sensor measurements enables the generation of reliable and comprehensive data for predicting the long-term evolution of installations and ultimately assessing disposal operation compliance.

Machine learning has emerged as an invaluable tool for THMC physical parameters’ forecasting [1,2], owing to its adeptness in handling intricate patterns and vast datasets. The spectrum of machine learning methods applicable to temperature forecasting is extensive, presenting a diverse array of techniques. This diversity brings us to a focus on the most prevalent methods employed in this domain. Linear regression models constitute a fundamental approach for straightforward temperature prediction tasks. These models establish a relationship between input features, such as historical temperature data, geographical information, and temperature forecasts [3]. Time series models, including the AutoRegressive Integrated Moving Average (ARIMA) [4] and Exponential Smoothing [5], stand as robust contenders for time series forecasting, catering to short-term temperature predictions.

Deep learning models currently dominate the landscape, in particular Recurrent Neural Networks (RNNs) and Convolutional Neural Networks (CNNs). RNNs, proficient in capturing temporal dependencies within time series, prove effective for short-term forecasting [6]. Conversely, Long Short-Term Memory (LSTM) Networks, a subset of RNNs, excel in modeling prolonged dependencies within time series data [7,8]. CNNs, adept at addressing spatial dependencies in physical parameters’ forecasting, find application in predicting physical parameters across geographical regions [9,10]. Additional established approaches encompass Random Forests [11], pooling multiple decision trees to enhance accuracy. Support Vector Machine (SVM) [12], applicable in the presence of non-linear and high-dimensional data, presents another way for physical parameters prediction. Moreover, Gaussian Process models [13,14] provide a means to encapsulate uncertainty in the forecasting process, bringing out probabilistic predictions.

Lastly, hybrid models [15] constitute a smart and fit-for-purpose way to encompass the three fundamental components (introduced at the beginning) for long-term monitoring, involving a fusion of machine learning models with physical models to enhance prediction accuracy. The central focus of this paper lies in the hybrid approach, showcasing its potential for enabling “smart monitoring” of complex systems by leveraging both physical-based models (i.e., numerical simulations) and residual data-driven models (i.e., machine learning using sensor data).

The main objective of the study is to develop a hybrid twin demonstrator for an HLW cell demonstrator (ALC1605) located within Andra’s Underground Research Laboratory (URL), focusing on the thermal parameter. Throughout this research, we delve into the differentiation between “digital” and “hybrid” twin modeling approaches. The term “digital twin” is utilized to describe the digital replication of physical assets, derived from real-life data collected through various sensors and monitoring technologies while the asset is in operation. This process typically involves creating an analytical, data-driven model (referred to as the twin) to analyze, update, and manage the performance of its physical counterpart. In contrast, the term “hybrid twin” is associated with solutions that involve constructing an additional, complementary virtual model. This supplementary model is inherently physics-based and delineates cause-and-effect relationships. This initiative encompasses a fusion of numerical simulation, real structure measurements, and data mining techniques, including both machine learning and deep learning.

The paper is structured as follows: In Section 2, we provide a detailed description of the ALC1605 instrumented heating cell. In Section 3, we expound upon the methodology used to construct the hybrid twin model and its constituent components. Section 4 is devoted to the evaluation of the model’s performance, featuring illustrative examples. Section 5 explores the practical industrial applications of this methodology. To finish, Section 6 offers some key insights derived from the research presented in this paper.

## 2. The ALC1605 Experiment: Sensors and Raw Data Quality

**Figure 1:**
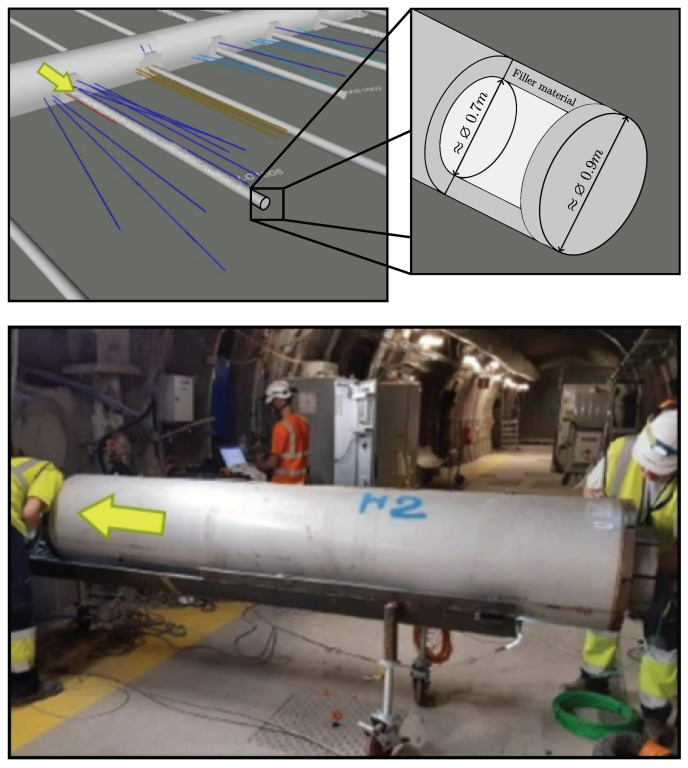
URL 3D BIM extract showing the ALC1605 demonstrator (**top**) and picture showing the introduction of one of the 5 heating probes (**bottom**). The green arrows show the direction in which the heating probes are introduced.

The ALC1605 experiment, conducted at Andra’s Underground Research Laboratory (URL), is an in situ heating test aiming at assessing a high-level waste (HLW) cell concept. It consists of a horizontal micro-tunnel with an excavated (drilled) diameter of approximately 0.9 m and a steel casing of 0.7 m (a specific filling cement material is placed in between the outside of the casing and the rock). Figure 1 shows the concept of the experiment, with the 25 m long micro-tunnel and surrounding boreholes for Thermo-Hydro-Meca (THM) measurements. The ALC1605 HLW cell demonstrator uses heating resistors to reproduce the rise in temperature as it will appear in the presence of the HLW. The general objective of the demonstrator is to improve the representation of the impact of thermomechanical loading on the cell (on the steel casing, on the rock, and on the filling material placed in between the casing and the host rock). Figure 1 illustrates the overall dimensions of the tunnel, which has a diameter of approximately 0.9 m. Inside the tunnel, a steel casing is placed, with a diameter of around 0.7 m. The annular space between the outer surface of the casing and the rock is filled with a a cementitious material. The heat probes, with a diameter of approximately 0.56 m, are then placed inside the casing. As it is highly instrumented with hundreds of sensors (including distributed and point measurements), it represents a good opportunity to implement and test digital twin technologies.

**Figure 2:**
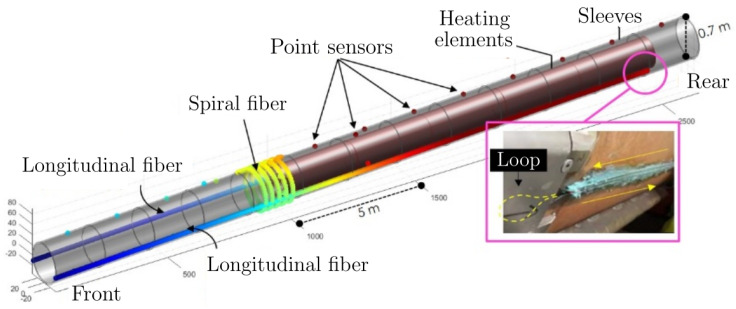
Schematic representation of the ALC1605 experiment, showing the main components and the monitoring configuration of the HLW cell demonstrator. The loop created by the return of the horizontal longitudinal optical fiber sensors is highlighted with a pink box.

The ALC1605 monitoring configuration is illustrated in Figure 2. This setup combines point and distributed sensors to acquire THM measurements continuously. Since the aim of this experiment is to model the behavior of the HLW cell over long periods, we stored one measurement per day. For this work, we focus on the thermal measurements from the THM data. Thermal data are collected using various conventional sensors such as Platinum RTD probes (PT100 and PT1000), thermocouples, and optical fibers (Raman-DTS). Using optical fibers for distributed measurements presents several advantages, such as enhanced spatial resolution, broader coverage, simultaneous measurement of multiple parameters, and potentially greater cost-effectiveness compared to point measurement systems, particularly when considering the same volume of data. The most costly component, the optical fiber interrogator, is placed outside the HLW cell. Distributed sensing is also particularly beneficial for comprehending complex systems or phenomena requiring detailed spatial insights and real-time monitoring. Moreover, optical fibers offer numerous advantages in harsh environments and have been extensively studied in this regard [16,17,18]. In this experiment, several optical fibers were deployed longitudinally outside the casing (illustrated in Figure 2) to examine the horizontal distribution and changes in thermal loading. To ensure redundancy in the measurements, each longitudinal optical fiber sensor was installed with a return at the end of the cell, creating the loop highlighted with the pink box in Figure 2. Additionally, as shown in Figure 2, optical fibers were spiral-wrapped around the steel casing to enhance the coverage of the cylinder’s surface, providing insights about the radial distribution of the thermal loading. Apart from the monitoring configuration, Figure 2 illustrates several elements of the HLW cell such as the sleeves or steel casing, which cover the whole tunnel, but are split into several sections to ease deployment, each with a length of approximately 2 m. The figure also shows the five heating probes used in the experiment to mimic the behavior of the HLW packages when the storage facility becomes operational. These heating probes have a length of approximately 3 m and a diameter of 0.56 m.

### Data Processing Strategy

Supervised machine learning approaches (such as standard multivariate regression) need to use both target and explanatory variables. However, in the context of missing data reconstruction (due to sensor failure), it is difficult to define explanatory and target variables in advance as undefined sensors may contain missing values in the future. An unsupervised method appears to be more suitable for this problem. Low-rank approximation of the dataset is one of the most widely used tools in unsupervised machine learning [19]. The rank of a matrix is considered low when it is small relative to the number of vectors that compose it. In the case of a smooth distribution of the thermal loading, we consider that the low-rank hypothesis is appropriate to the extent that a large portion of the data variability is explained with few components (or singular values) or at least a small number relative to the number of sensors.

To address anomalies observed in the raw data such as missing values, data corruption, and drifting, we tested two techniques aiming at capturing the low-rank structure of the data: Robust Principal Component Analysis (R-PCA) [20,21] and Singular-Value Thresholding (SVT) [22]. R-PCA is a method that splits a dataset into two parts: a low-rank part representing the core structure and a sparse part accounting for outliers or noise. Traditional PCA assumes clean data following a Gaussian distribution, but real-world data often contain outliers, impacting PCA’s effectiveness. R-PCA tackles this by employing robust statistical techniques to distinguish the low-rank structure from sparse outliers using optimization algorithms (the ADMM as detailed in [23]) to minimize the low-rank component’s rank while promoting sparsity in the sparse component. In scenarios like spatially smooth temperature distributions from multiple sensors, RPCA assumes the underlying structure can be represented by a matrix with few dominant patterns, especially when temperature changes smoothly across space. By decomposing data into low-rank and sparse components, R-PCA enables estimating missing values based on the data’s low-rank structure, making it valuable for data imputation tasks.

**Figure 3:**
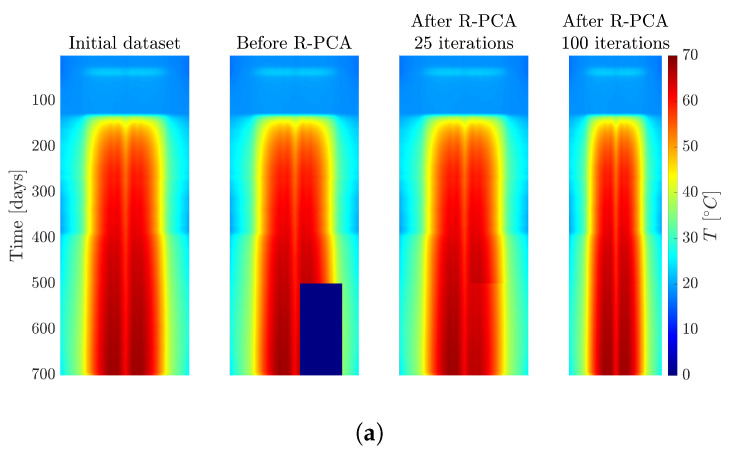

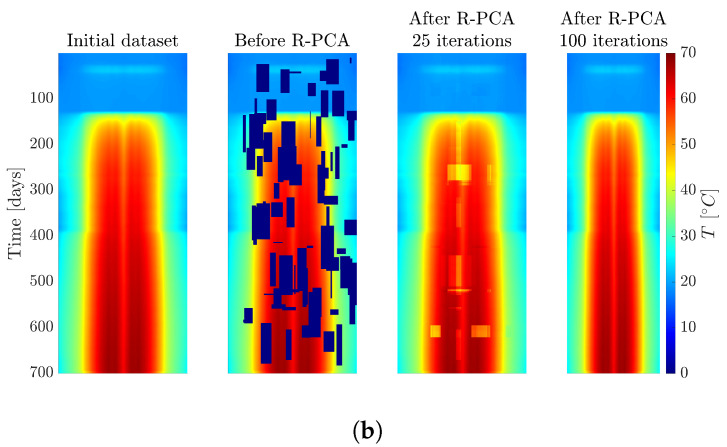
Synthetic test to evaluate the effectiveness of R-PCA in recovering missing data and correcting outliers. (**a**) R-PCA test involving the removal of a portion of the initial dataset. (**b**) R-PCA test where we randomly remove portions of the initial dataset.

SVT is a method used for missing data imputation, like R-PCA. It operates by shrinking singular values towards zero while preserving dominant ones through optimization. This captures a low-rank structure in the data, making it useful for estimating missing values while maintaining data integrity. Both methods were efficient in our case study and yielded similar results, with R-PCA being more practical for implementation on our system architecture. The raw data obtained directly from the sensors are organized in a tabular format, recording daily temperature readings for each sensor. Recognizing the time dependency inherent in the data, this tabular information can be transformed into an image, where the rows represent the temperature’s evolution over time and the columns represent different point measurements in the optical fiber sensor. With this transformation, we can then apply various techniques to the resulting image. For instance, Figure 3 considers a synthetic experiment conducted on the acquired data. In particular, Figure 3a takes the original image of measurements (left) and removes a large portion from it (middle-left). Then, the R-PCA algorithm is applied to the image with anomalies. The resulting images when the iterative process reaches 25 and 100 iterations are shown as well (middle-right and right, respectively), ultimately producing an error of about 2%. Similarly, Figure 3b repeats the experiment, but in this case, the portion of the image removed is randomly distributed over the whole image, instead of a large concentrated portion. This approach also yields errors of approximately 2%.

**Figure 4:**
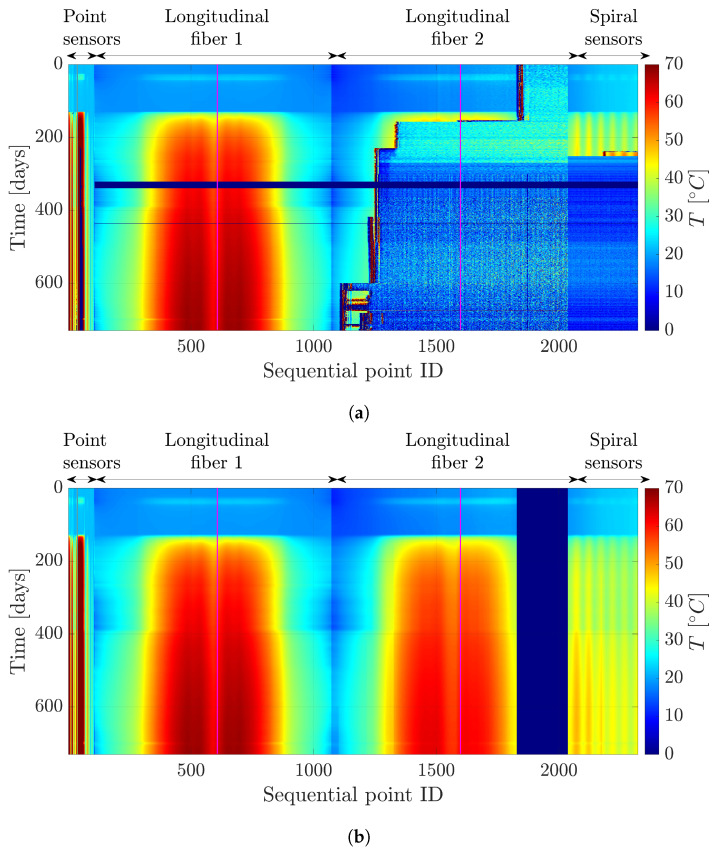
Comparison of the temperature dataset before (**a**) and after (**b**) applying the R-PCA technique. The labels correspond to those used in Figure 2, with the loop of the longitudinal fiber optic sensors highlighted in pink for clarity. (**a**) Original dataset that contains the measurements from the optic fiber sensor. (**b**) Reconstructed dataset employing the R-PCA technique over the measurements from the optic fiber sensors.

Once we have evaluated the suitability of the R-PCA technique in recovering synthetically altered data, we can apply it to the original dataset containing all measurements. Figure 4a displays the raw data in image format, highlighting two main anomalies: (1) regions with missing values and (2) regions with sudden temperature changes resembling noise. Upon applying the R-PCA technique (Figure 4b), we effectively mitigate the impact of missing data and reduce the influence of noise-induced temperature fluctuations in the measurements.

## 3. Methodology

This section outlines the methodology employed in developing the hybrid twin model for the ALC1605-instrumented heating cell. We will begin by delving into the operational principles of the hybrid twin model, followed by the analysis of its two key components: the physics-based model and the data-driven model.

### 3.1. Hybrid Twin Approach

A hybrid twin model aims to improve numerical simulations by integrating measurements obtained from the real system (i.e., the differences between the numerical simulation and the complex real system). In doing so, the hybrid twin model can take into account the subtleties inherent in the real system, which are difficult to simulate accurately. This integration leads to predictions that align more closely with the observed data. We define “residual gap” as the deviation between the real-world behavior and the outcomes produced by numerical simulations. Incorporating the residual gap model into these simulations enables us to faithfully replicate the actual application behavior, as depicted in the equation below:

(1) Y(X,t,μ)=A(X,t,μ)+B(X,t,μ),

where *Y* represents the observed real-world phenomena, while *A* denotes the physics-based solution, and *B* symbolizes the realm of uncertainty or the residual gap. Additionally, *X* signifies the spatial coordinates, *t* represents temporal values, and μ encompasses any additional parameters, including material properties, boundary conditions, and other relevant factors.

#### 3.1.1. Physics-Based Model

The physics-based model plays a critical role in incorporating the fundamental principles of physics into our application. In the context of the ALC1605 experiment, we leverage the Finite-Element Method (FEM) to model the temperature distribution within our system.

**Figure 5:**
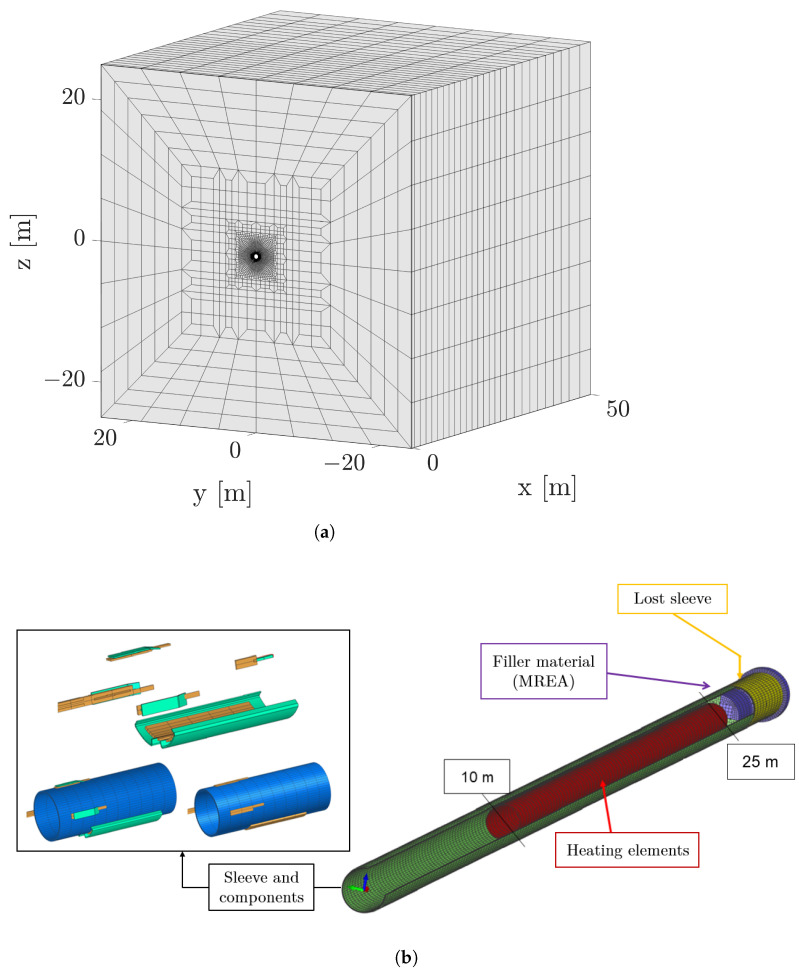
Overview of the different components employed to solve the finite-element calculations. (**a**) Finite-element model illustrating the domain surrounding the tunnel, excluding the sleeves. It includes the following materials arranged from inner to outer layers: filler material (MREA), fractured COx claystone delineating the connected fractured zone (ZFC) and the diffuse fractured zone (ZFD), and COx claystone. (**b**) Finite-element model illustrating the tunnel, primarily composed of heating elements and sleeves that partition the surrounding rock from the heating elements. Also depicted is the “lost sleeve”, a section of the tunnel completely filled with MREA instead of housing a heating element, along with small components positioned outside the sleeves to aid in their placement.

The components, the underground tunnel, and its surroundings are modeled using CAD tools and discretized into quadratic elements to facilitate the FE analysis. A visual representation can be found in Figure 5, which showcases the meshes utilized for this purpose.

In addition to calculating the thermal behavior of the domain through a conventional finite-element approach, the intricate interplay between the surrounding air, the sleeves, and the heating elements is ascertained by solving a diffusion–convection problem using one-dimensional elements in the heat transfer boundaries to precisely capture the heat exchange dynamics. This combined methodology ensures a robust and accurate representation of the complex thermal processes at play in the system, facilitating an understanding and effective analysis of the model’s behavior.

Material properties play an important role in the physics-based modeling. While the sleeves and their associated components exhibit material properties consistent with steel, the surroundings of the tunnel showcase diverse material characteristics in different regions. Even in the region adjacent to the tunnel, we can delineate three distinct zones, each governed by unique material properties: the filler material, referred to as MREA, the connected fractured zone, abbreviated as ZFC, and the diffuse fractured zone, referred to as ZFD. This differentiation stems from the impact on the adjacent zone during borehole drilling, resulting in modified material properties, particularly a decrease in thermal conductivity. Furthermore, it is crucial to note that the thermal conductivity of these regions is anisotropic, dependent on the rock’s stratification layers [24]. To provide a detailed description of the model, we have included the specific values of each material and their physical properties in Table 1.

Hence, for the purposes of this project, rather than having a physics-based model that relies solely on a fixed set of parameters, we are more interested in an adaptable and versatile parametric solution that takes into account real-time constraints and can accommodate changes in the input parameters. The first step in creating such a parametric solution is the definition of the parameters that we need to explore. By considering the variations and bounds of these parameters, the multidimensional space that we intend to explore can be defined, and we can conduct analyses and simulations to generate corresponding temperature fields. These temperature fields provide an understanding of the system’s behavior under different parameter settings, allowing us to adapt and optimize our solutions in response to dynamic real-world conditions.

Once the bounds of the parameters have been defined and the simulations that are necessary to explore the parameter space have been performed, the temperature fields that we obtain can be used to construct a surrogate model, which is a mathematical model that can approximate the temperature field given a new set of parameters. To achieve this, we employ a systematic approach. Firstly, we organize the collected temperature data into a structured matrix ($A_{i}$), for a given set of parameters. Next, using the CUR decomposition [25], we extract and represent the essential components of the data. In the context of this decomposition, careful consideration has been given to the selection of matrices to maintain the constancy of C and R, irrespective of the chosen parameters, while matrix $U_{i}$ is intentionally designed to dynamically adapt based on the specific parameter values. Consequently, we obtain the following expression for a given parameter set:

(2) $$A_{i}=C\cdot U_{i}\left(P_{1},P_{2},\ldots ,P_{N}\right)\cdot R^{T}.$$

By utilizing the regressions based on the Sparse Proper Generalized Decomposition (sPGD) framework [26], the matrix $U_{i}$ can be derived for any set of parameter values. This approach enables rapid “on the fly” testing of various parameter configurations in real time, eliminating the need for conducting heavy and time-consuming finite-element calculations. It offers two key advantages: first, it removes reliance on a specific simulation platform, and second, it allows for swift execution of parameter configuration tests, even on a standard laptop.

**Figure 6:**
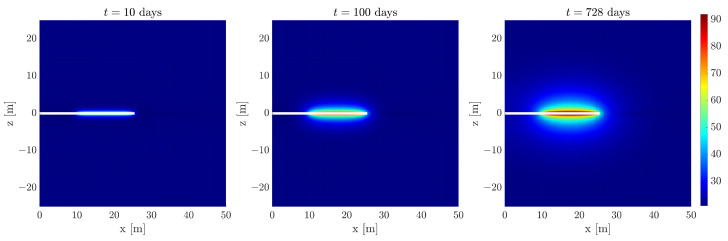
Progression of the temperature distribution over time, as influenced by a specific set of parameters.

Figure 6 presents a visual representation of the dynamic evolution of the temperature field in a given scenario. In this scenario, we consider that the thermal conductivity of the MREA is 0.89 W/(m·K), while the horizontal and vertical thermal conductivity of the ZFC are 1.64 and 1.06 W/(m·K), respectively. The heating elements are assumed to operate at a power level of 675 watts per element, while a uniform temperature of 21 °C is imposed across the entire system as an initial condition. The external surfaces of this system are assumed to be adiabatic, signifying no heat exchange with the surroundings.

The essence of the hybrid twin approach lies in augmenting the physics-based solution with the available measurements at our disposal. Subsequently, the following section will explore the modeling of the disparities between the employed physics and the actual behavior observed through sensor data.

#### 3.1.2. Data-Driven Model

The data-driven model under development plays a crucial role in representing a concept commonly referred to as “residual gap”. To address this, we adopt the residual neural networks (ResNets) architecture [27,28,29], recognized for its effectiveness in capturing complex temporal dependencies. ResNets offer a powerful framework for establishing a robust methodology based on well-established temporal integration techniques, notably the forward Euler scheme [30,31], as explained in Equation (3).

Our approach involves integrating Long Short-Term Memory (LSTM) cells within the ResNet architecture. The decision to use the LSTM deep learning architecture stems from its outstanding performance in handling sequential data. LSTM cells, seamlessly integrated into our model, provide an evanescent memory for the long-term path and a combination of long and short leads for the short-term memory response. By leveraging these characteristics, our primary objective is to craft a highly effective model capable of capturing the temporal dynamics inherent in the data sequences and harness the strengths of both ResNets and LSTM cells.

(3) $$B_{t}=\frac{\partial B_{t}}{\partial t}\cdot \Delta t+B_{t-1}.$$

In the context of our hybrid twin framework, the symbol *B* is used to represent the variable associated with the element of residual gap. The subscripts t−1 and *t* signify two consecutive time steps. The essence of our methodology aims to model the rate of change of residual gap over time. As a first step in this process, we seek to replace it with an unknown function, denoted as *H*, resulting in the following transformation:

(4) $$B_{t}=H\left(B_{t-1},\mu_{t}\right)\cdot \Delta t+B_{t-1},$$

where function *H* is influenced not only by the current residual gap, denoted as $B_{t-1}$, but also by additional parameters represented by $\mu_{t}$. These supplementary parameters encompass spatial coordinates, ambient temperature evolution, and material properties, all of which are crucial components integrated into the model. Furthermore, the additional parameters may also encompass the present state of the physics-based model $A_{t}$. In a similar vein, the function *H* can be substituted with a combination of two distinct functions, namely *f* and *g*, expressed as follows:

(5) $$H=f\left(B_{t-1},\mu_{t}\right)\cdot B_{t-1}+g\left(\mu_{t}\right),$$

The functions *f* and *g* serve as the components that the neural network (NN) will model. Employing this approach is crucial to guarantee the stability of the temporal integration. Specifically, by constraining the values of the *f* function to be below 0, we aim to prevent the divergence of temporal integration [32]. This constraint is strategically implemented to ensure a robust and reliable performance of the NN in capturing and processing temporal dependencies. The NN architectures considered for describing functions *f* and *g* are both based on the use of LSTM layers combined with a deep dense neural network layer, as described in Table 2 and Table 3, for ResNet. They were built by using Tensorflow Keras libraries.

**Figure 7:**
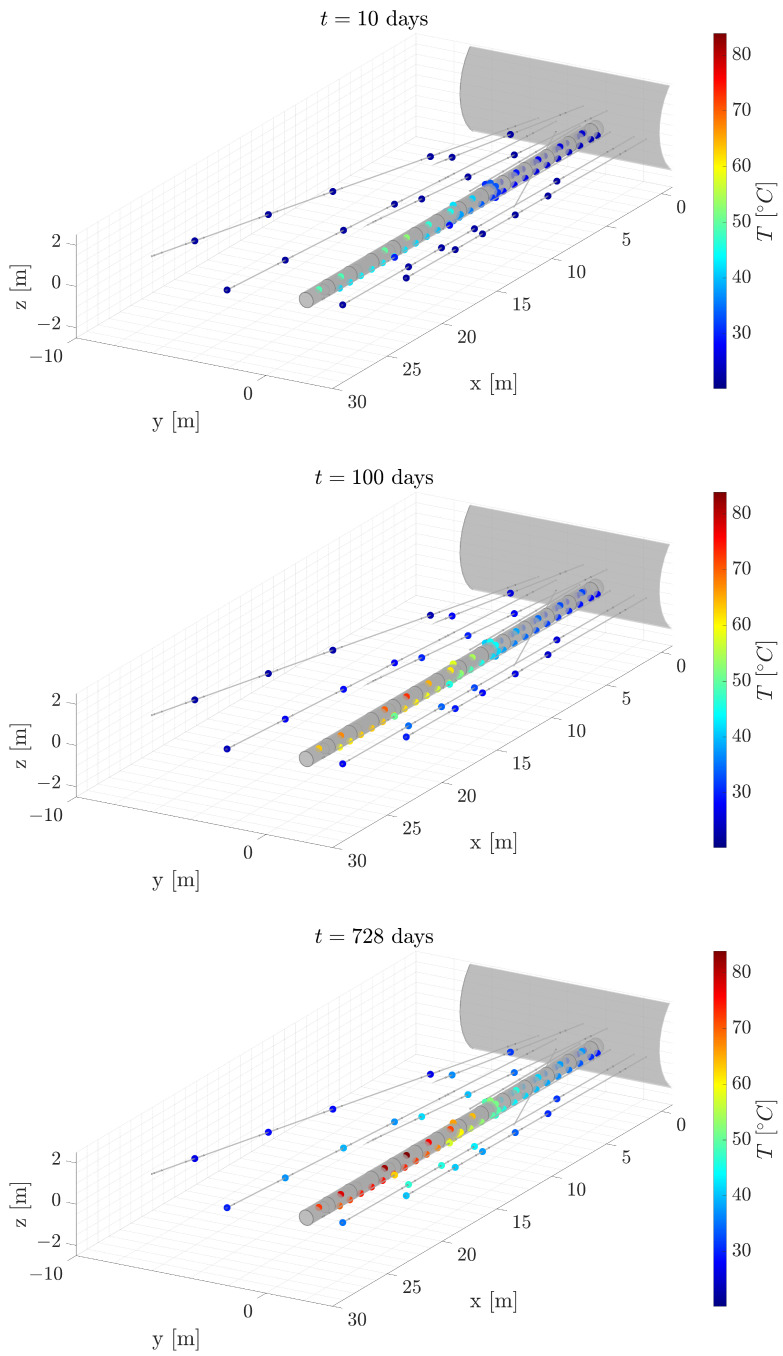
Temperature measurements taken at three distinct time steps for each sensor.

Once the model has been defined, the next step is to train it. However, before initiating the training process, it is imperative to preprocess the data to ensure their compatibility with the LSTM architecture. In their raw form, the data collected from the sensors typically resemble a tabular structure that encapsulates the readings from each sensor at various time intervals. To illustrate this concept visually, consider Figure 7, which presents a graphical representation of the sensor data. In this figure, you can observe the temperature readings recorded by each sensor across three distinct time steps. This representation offers a clear visualization of how the data are organized, with each sensor’s temperature measurements evolving over time.

**Figure 8:**
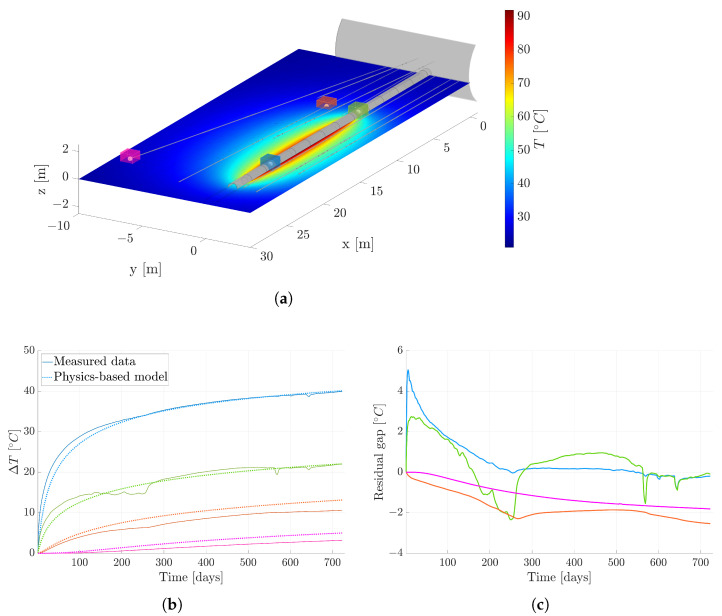
Position and evolution curves for the four reference sensors. (**a**) Position of the four reference sensors. (**b**) Temperature evolution for the four reference sensors. (**c**) Residual gap evolution for the four reference sensors. The colors of the curves correspond to the sensors illustrated in (**a**).

To prepare the dataset for our model, it must be transformed into sequential data. Before proceeding, we establish four reference sensors. These sensors will be intentionally excluded throughout the modeling process to evaluate the final efficacy of the proposed methodology. Figure 8a illustrates the positions of these three selected sensors, along with a section of the physics-based numerical solution from the final time step. The selection of these sensors is meaningful as it allows us to evaluate the methodology across various contexts:

- Purple sensor: Positioned in a region extremely distant from the heaters, where accurately characterizing the rock’s conductivity is crucial.
- Orange sensor: Located in a region distant from the heaters, where the drilling of the tunnel has affected the rock’s physical properties.
- Blue sensor: Situated near the tunnel walls, where the interaction between the air and the heaters poses a modeling challenge.
- Green sensor: Placed adjacent to the gallery, influenced by external temperatures and their interplay with the tunnel environment.

We anticipate that the data-driven model will perform well in regions where the physics-based model exhibits shortcomings, namely areas characterized by complex modeling due to uncertainties in physics couplings or material properties. Additionally, Figure 8b portrays the temporal evolution of measured temperatures for this trio of reference sensors. To enrich the visual depiction, temperature data generated by the physics-based model at corresponding positions are also included. Leveraging Equation (1), we can ascertain the level of uncertainty denoted as “residual gap”, represented by the variable *B*. This uncertainty is determined by subtracting the solution derived from physics-based modeling from experimentally measured temperatures. The temporal evolution of this uncertainty is depicted in Figure 8c, pertaining to the same set of sensors.

**Figure 9:**
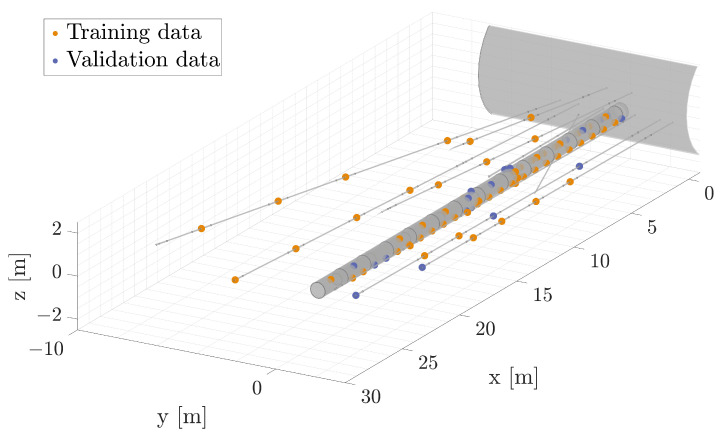
Detail of the sensor partition considered for training the model.

Once we have successfully defined the data, we are ready to proceed with training the model. The initial step in this process involves the proper partitioning of the data. We start by allocating the first 120 time steps for training and validation, reserving the subsequent steps for performance evaluation. This phase, referred to as the calibration training period, can be adjusted to meet specific requirements. Once calibrated, our model not only demonstrates predictive capabilities, but also excels at detecting and signaling anomalies, such as sensor drift or physical changes, thus enhancing its utility in real-world applications. Within the temporal dataset, encompassing the initial 120 time steps, we execute a partitioning operation to create distinct subsets for training and validation. This partitioning is accomplished with a specific split ratio of 80% for training and 20% for validation, as shown in Figure 9. This strategic division allows us to effectively evaluate the model’s performance and refine its predictive capabilities.

The training of a machine learning model involves selecting a large number of hyperparameters that significantly affect the model’s final performance. In this work, some hyperparameters are set directly, while others are determined algorithmically. The directly set hyperparameters include the activation functions used in the various neurons of our model, as detailed in Table 2 and Table 3. Additionally, we employ the Adam optimizer [33] to adjust the weights and biases based on the Mean-Squared Error (MSE) loss function. We set the maximum number of epochs to 200 and use an adaptive learning rate that starts at 0.05 and decreases as training progresses. The remaining hyperparameters are determined algorithmically, as explained below.

When considering hyperparameters that influence the definition and training of a machine learning model, several key aspects deserve special attention. The hyperparameters we consider encompass the ultimate size of the input sequence data, which can often be judiciously reduced to enhance flexibility in predictive capabilities. Additionally, the number of units residing within the LSTM cells plays a pivotal role in determining model complexity and performance. Further, the architecture’s overall structure might entail auxiliary layers, necessitating careful calibration of the number of neurons in these layers to accommodate potential disparities in the sizes of the primary layers. The incorporation of the $L_{2}$ regularization term is crucial for mitigating overfitting, ensuring the model’s generalizability and preventing excessive complexity. Lastly, one must fine-tune the batch size, a parameter that significantly influences the training dynamics, convergence speed, and memory requirements of the model. Therefore, meticulous selection and tuning of these hyperparameters are fundamental in the quest for effective and efficient machine learning models.

We trained a model with a specific set of hyperparameters, enabling subsequent queries. This allowed us to make forecasts over time by integrating Equation (4) into a closed-loop system, resulting in:

$$\begin{array}{c} B_{t}=H\left(B_{t-1},\mu_{t}\right)\cdot \Delta t+B_{t-1}\\ B_{t+1}=H\left(B_{t},\mu_{t+1}\right)\cdot \Delta t+B_{t}\\ \vdots \\ B_{T}=H\left(B_{T-1},\mu_{T}\right)\cdot \Delta t+B_{T-1} \end{array}$$

**Figure 10:**
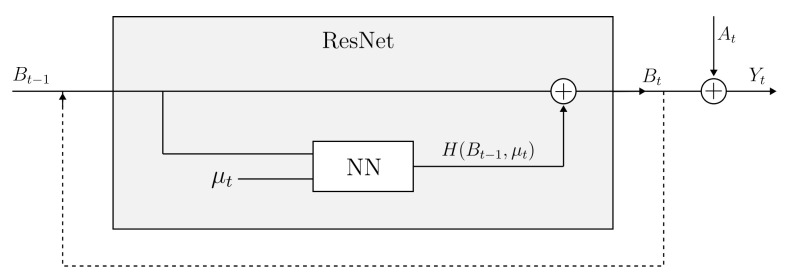
Temporal integration scheme of the hybrid twin approach.

In the forecasting stage, we integrate insights from physics-based numerical simulations into our data-driven model. This fusion enhances the simulation’s performance, enabling it to closely replicate real-world phenomena. The complete time integration scheme it is represented in Figure 10.

After forecasting the temperature, it is essential to assess the performance of the associated model. To accomplish this, we establish a set of error metrics. Initially, we define the error for a particular sensor at a specific time step as follows:

(6) $$\varepsilon_{s,t}={\left(Y\left(X_{s},t\right)-\hat{Y}\left(X_{s},t\right)\right)}^{2},$$

where *Y* denotes the measured temperature and $\hat{Y}$ represents the predicted temperature. In this context, *s* serves as an indicator for a specific sensor and *t* represents an individual time step. Consequently, we define the error for a particular sensor as the average of the sum of errors across all time steps, which can be expressed as follows:

(7) $$\varepsilon_{s}=\frac{1}{T}\sum_{t=1}^{T}\varepsilon_{s,t}.$$

Finally, the model’s error is assessed by calculating the average summation of errors across all sensors, where each sensor’s error is considered, as follows:

(8) $$\varepsilon_{m}=\frac{1}{S}\sum_{s=1}^{S}\varepsilon_{s}.$$

The determination of the optimal hyperparameter configuration relies on identifying the model with the lowest value of $\varepsilon_{m}$. The following hyperparameter settings were employed for the subsequent results:

(a) The length of the temperature sequence for training and validation was fixed at 16 elements.
(b) The dimensionality of the hidden state in the LSTM layer was set to 2.
(c) The number of neurons in the dense layer was configured to 16.
(d) $L_{2}$ regularization with a coefficient of $1\times 10^{-4}$ was applied to the weights and biases.
(e) The batch size utilized during training was 128.

These parameter selections were crucial in achieving optimal performance and ensuring robustness in the model’s training and validation phases.

## 4. Experimental Results

This section is dedicated to showcasing the robust capabilities of the proposed methodology, which leverages the hybrid twin approach, in the context of forecasting temperature within a radioactive waste disposal facility. The insights and findings presented herein are derived from an exhaustive model evaluation process, where various hyperparameters were rigorously tested and refined. Among this extensive exploration, we identified the specific set of hyperparameters that yielded the lowest error, thus ensuring the utmost accuracy and reliability in our temperature forecasts.

**Figure 11:**
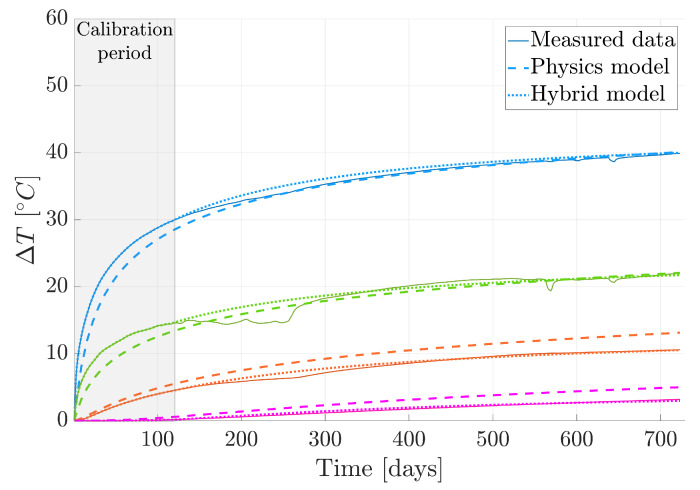
Temperature evolution from the measured data, physics-based solution, and hybrid twin approach. The colors of the curves correspond to the sensors illustrated in Figure 8a.

To evaluate the model’s performance, we reintroduce the four sensors excluded from the original dataset. Figure 8a displays the positions of these reference sensors in space. Additionally, Figure 11 provides a visual representation of temperature changes over time. Three types of curves are depicted: solid lines represent the actual measured temperatures; dashed lines represent temperatures predicted by our physics-based model; dotted lines represent temperatures predicted by our hybrid approach.

Upon analyzing these curves, we observed a significant enhancement in forecasting accuracy resulting from integrating the data-driven model into the physics-based one. This underscores the advantage of adopting a hybrid approach rather than relying solely on physics-based methodologies. The integration effectively combines the strengths of both data-driven and physics-based approaches, resulting in more reliable predictions.

**Figure 12:**
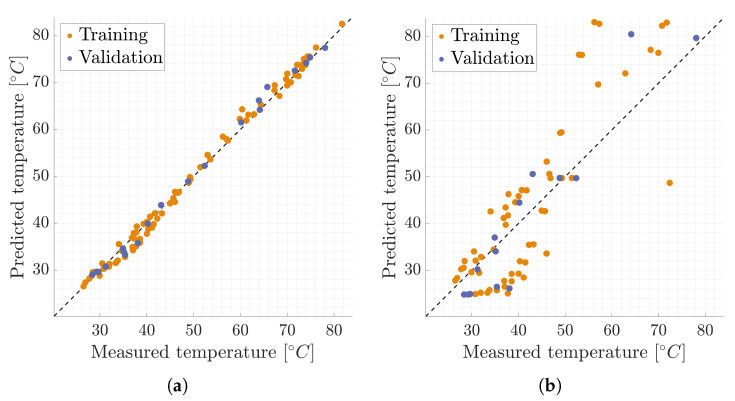
Comparison between the measured temperature and the forecasted temperature for the last time step. The dashed line represents y=x. (**a**) Hybrid twin model. (**b**) Physics-based model.

To obtain an understanding of the model’s performance on a global scale, Figure 12a illustrates the comparison between the measured temperature and the forecasted temperature utilizing the hybrid twin approach, specifically for the final time step. This particular visualization holds particular significance due to the accumulation of errors over the course of the temporal integration process. The last time step can offer invaluable insights into the model’s behavior, encapsulating the culmination of the forecasting performance, making it a critical focal point for evaluation and analysis.

To properly assess the model’s performance, we must revisit an event from the data capturing stage. Upon examining the green solid line in Figure 11, representing data from a specific sensor, we notice a sudden temperature increase between days 200 and 300. This anomaly correlates with the closure of the gallery gate, indicating an external influence on the measurements.

Subsequently, the measurements appear affected by this event, marked by a significant temperature rise. It is crucial to recognize that this deviation from expected behavior does not reflect the model’s intended performance. Hence, the deliberate choice to use the initial 120 time steps for training aims to mitigate such anomalies.

Despite this deviation, valuable insights can be gleaned from this anomalous behavior. The proposed methodology not only establishes a baseline model for the expected performance of the tunnel and its surroundings, but also possesses the ability to alert when a sensor displays abnormal behavior due to drifting measurements. Thus, this nominal model not only sets a benchmark, but also serves as a tool to identify deviations from expected sensor performance.

However, we now have the opportunity to compare the outcomes generated by the hybrid twin model with those exclusively reliant on physics. This comparison aims to determine if a notable enhancement has been achieved. To facilitate this evaluation, we present the error graph for the physics-based solution in Figure 12b. The visual representation clearly demonstrates a significant improvement in overall performance when incorporating additional data-driven insights. This underscores the concept that the precision of the physics-based solution does not necessarily need to be exceptionally high; instead, it can be substantially elevated through the integration of data. This inherent capability has the potential to considerably streamline the modeling process, meshing activities, and calculations, ultimately reducing the time investment required.

## 5. Application Usage

The preceding section has demonstrated the potential of the proposed hybrid twin approach in forecasting temperature fields, showcasing its applicability across various industrial scenarios. Of particular significance are its applications in sensor diagnosis, as previously mentioned, and its capacity to extrapolate temperature data across all spatial coordinates within the domain, extending beyond sensor placement locations.

### 5.1. Sensor Diagnosis

Our system has a practical application in diagnosing and monitoring sensor performance. To demonstrate, let us revisit our database where certain sensors faced disruptions in a laboratory setting. This was caused by the sudden closure of a security gate, resulting in a rapid temperature rise. It is important to note that such events can vary, from gate closures to sensor malfunctions.

**Figure 13:**
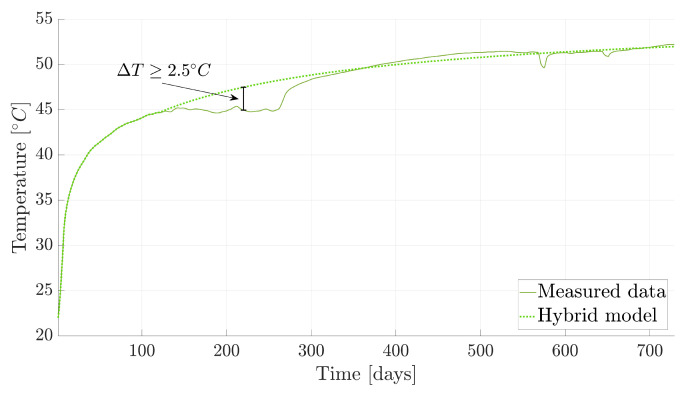
Temperature evolution from the measured data and hybrid twin approach for a sensor suspected of malfunctioning.

Our approach to diagnosing malfunctioning sensors involves two stages. First is using hybrid twins to model expected sensor behavior. Any deviation from this expected behavior serves as an early warning of potential malfunction. Taking the example of the green sensor (Figure 8a), we observe the temperature evolution in Figure 13. The measured data show a temperature stagnation followed by a sudden increase between days 100 and 300, while the hybrid twin model maintains the expected monotonic behavior. By comparing both and setting an error threshold (ΔT≥2.5 °C, for instance), we can identify instances of data drift, indicating a potential sensor issue.

Once we can identify the malfunctioning sensor, it becomes the responsibility of the operator to thoroughly evaluate the situation, as the measured data or hybrid twin model cannot definitively determine the cause of the problem.

### 5.2. Domain Completion

In this section, we highlight a significant industrial application: the expansion of the improved solution across the entire domain, extending beyond sensor locations to ascertain accurate temperatures throughout the rock.

Our strategy hinges on a conventional Proper Orthogonal Decomposition (POD) approach [34,35]. Specifically, we utilize a temperature matrix derived from the physics-based solution, where rows denote nodes and columns represent time steps. We decompose this matrix using the following equation:

(9) $$A=V_{A}\cdot \Sigma_{A}\cdot W_{A}^{T}=V_{A}\cdot \alpha_{A}.$$

By selecting a limited number of POD modes, we reconstruct an approximate version of the original matrix as follows:

(10) $$A\approx V_{A}^{r}\cdot \alpha_{A}^{r}.$$

Moreover, the temperature predicted by the hybrid twin model at the sensor’s location can be initially represented as a matrix ($\hat{Y}$), which can subsequently be decomposed. It is crucial to emphasize that, in this scenario, the matrix exclusively comprises sensor data, as these comprise the available and known information (denoted by $\hat{\cdot }$).

**Figure 14:**
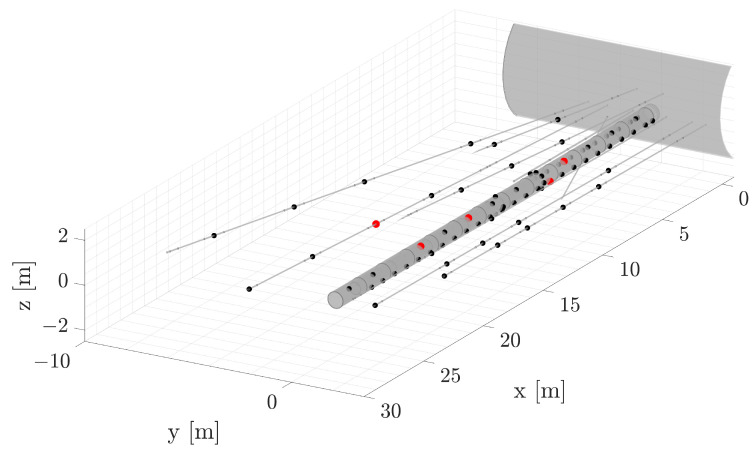
Detail of the sensor selection (in red) employed for the POD completion strategy, contrasted with the other sensors (in black).

However, incorporating all available sensors may introduce noise into the strategy, which could impact the selection of the number of POD modes. Ideally, we aimed to minimize this number to avoid high-frequency modes, which are not aligned with the expected behavior. Therefore, the selection of sensors utilized for these results is depicted in Figure 14, highlighting the selected sensors in red.

Additionally, $V_{A}^{r}$ represents a basis composed of a set of orthogonal modes defined in the domain. These modes can be utilized to derive the corresponding set of vectors, $V_{\hat{A}}^{r}$, at the sensor’s location. Consequently, the decomposition of the matrix $\hat{Y}$ follows the expression:

(11) $$\hat{Y}\approx V_{\hat{A}}^{r}\cdot \alpha_{\hat{Y}}^{r}\to \alpha_{\hat{Y}}^{r}={\left(V_{\hat{A}}^{r^{T}}\cdot V_{\hat{A}}^{r}\right)}^{-1}\cdot \left(V_{\hat{A}}^{r^{T}}\cdot \hat{Y}\right)$$

where the unknown variables are represented by the coefficients $\alpha_{\hat{Y}}^{r}$ and may be obtained by solving the resulting system of linear equations. The final step involves utilizing the recently acquired coefficients to extend our understanding across the entire domain through the use of the matrix $V_{A}^{r}$, as follows:

(12) $$Y\approx V_{A}^{r}\cdot \alpha_{\hat{Y}}^{r}.$$

**Figure 15:**
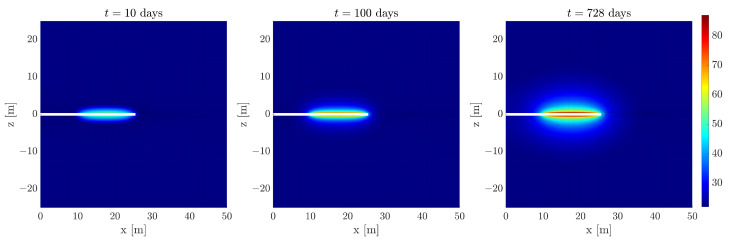
Progression of the temperature distribution over time when the enhanced solution in the sensors is extrapolated to the whole domain. It is worth noting that, for comparison purposes, the color bar used corresponds to that of the physics-based model.

This completion method yields the temperature fields, as illustrated in Figure 15, for three time steps. These results were obtained employing four POD modes and the selection of sensors shown in Figure 14.

**Figure 16:**
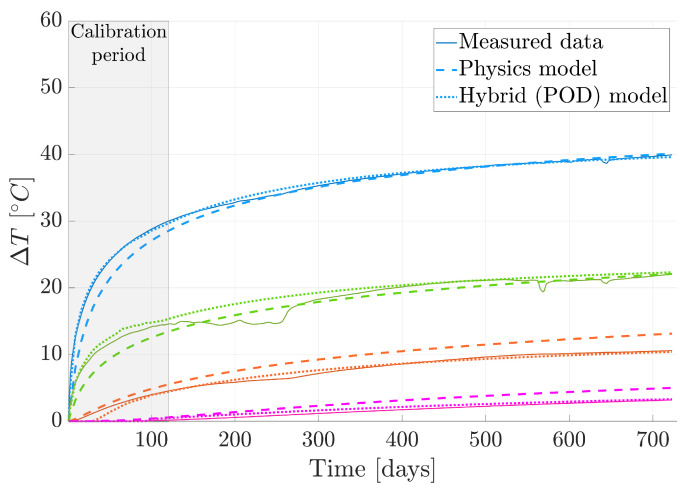
Temperature evolution is shown here from measured data, a physics-based solution, and the hybrid twin approach. The results presented for the hybrid twin model incorporate the domain completion strategy based on the POD technique. The colors of the curves correspond to the sensors illustrated in Figure 8a.

This method offers a tool that enables querying any point within the domain and provides the enhanced temperature through the hybrid twin model. To assess the performance of this strategy, we revisit the selection of the four reference sensors and request the corresponding temperature evaluation. The results are depicted in Figure 16.

The conclusions derived from applying the hybrid twin model are reiterated here, highlighting a significant enhancement compared to the physics-based model. This enhancement is evidenced by the close alignment between the proposed curves and the measured data.

## 6. Conclusions

Utilizing data from sensors in an HLW cell demonstrator at Andra’s underground research laboratory, we have proposed and successfully implemented a comprehensive digital methodology named “hybrid twin”. This approach integrates advanced machine learning and deep learning techniques with traditional physics-based modeling to address challenges in comparing sensor data to numerical simulations for monitoring complex real-world systems.

The hybrid twin methodology effectively merges the strengths of simulation data (offering spatial continuity and temporal predictive capabilities) with in situ sensor data (which, while localized and non-predictive over time, accurately represents the real system). This synergy enhances the monitoring and forecasting of the cell demonstrator’s evolution in both time and space.

Our methodology provides several key benefits: It improves long-term forecasting accuracy by grounding predictions in well-established physical principles, thereby enhancing the reliability of the results. This robust framework allows for a more controlled and precise comparison of in situ sensor data with well-calibrated numerical simulations, offering significant value for the monitoring of complex and real systems.

## Figures and Tables

**Table 1:** Physical properties of the different materials employed in the finite-element model.

Material	Thermal Conductivity [W/(m·K)]	Specific Heat Capacity [J/(Kg·K)]	Density [${\mathrm{Kg}/m}^{3}$]
Steel (sleeves)	50	460	7800
MREA	0.89	2560	1160
COx claystone	2.05 (horizontal) 1.33 (vertical)	800	2400
ZFC	1.64 (horizontal) 1.06 (vertical)	800	2400
ZFD	1.99 (horizontal) 1.29 (vertical)	800	2400

**Table 2:** The building blocks that model the function *f*.

Layer	Building Blocks	Activation
1	LSTM layer, hidden size = 2	sigmoid + tanh
2	Flatten	no activation
3	Dense layer, #neurons = 16	tanh
4	Dense layer, #neurons = sequence size	ReLU
5	Lambda layer returning − 1 × inputs	no activation

**Table 3:** The building blocks that model the function *g*.

Layer	Building Blocks	Activation
1	LSTM layer, hidden size = 2	sigmoid + tanh
2	Flatten	no activation
3	Dense layer, #neurons = 16	tanh
4	Dense layer, #neurons = sequence size	linear

## Data Availability

The participants of this study did not give written consent for their data to be shared publicly, so due to the sensitive nature of the research supporting data is not available.
